# MEMS Data-Driven Intelligent Identification of Rotation-Angle Response and Shear Band Position in Gravelly Soil Slopes

**DOI:** 10.3390/s26154871

**Published:** 2026-08-02

**Authors:** Di Wu, Yongzhe Feng, Gurong Yao, Hualin Song, Zhiwen Lu, Ming Wu

**Affiliations:** 1 School of Architecture and Transportation Engineering, Guilin University of Electronic Technology, Guilin 541004, China; wudi@guet.edu.cn (D.W.); songhualincn@gmail.com (H.S.); 2 Geological Environment Monitoring Station of Guangxi Zhuang Autonomous Region, Nanning 530201, China; 3 Department of Computer Science, KU Leuven, 3001 Leuven, Belgium; ming.wu1@kuleuven.be; 4 Department of Mechanical Engineering, KU Leuven, 3001 Leuven, Belgium

**Keywords:** gravelly soil slope, MEMS sensor, rotation-angle response, shear band identification, DEM simulation, machine learning

## Abstract

Identifying shear band locations is essential for precursor recognition and early warning of progressive failure in gravelly soil slopes. However, conventional displacement monitoring methods mainly capture macroscopic slope deformation and remain limited in detecting internal localized deformation and the spatial evolution of shear bands. To address this limitation, this study proposes an MEMS data-driven framework for predicting spatial rotation-angle responses and locating potential shear bands in gravelly soil slopes, with the aim of enhancing the perception of internal shear deformation and detecting potential instability zones. First, scaled laboratory model tests were conducted under different gravel contents, and MEMS sensors were embedded within the slope to measure cumulative rotation-angle responses during shear band formation. Second, based on a DEM model incorporating particle geometric morphology, the spatial differentiation of the rotation-angle field during shear band evolution was analyzed, and the experimental results were further validated. Finally, a shear band localization framework integrating PDL-GAN-based data augmentation with PCA + Gaussian regional rotation-angle field prediction was established. Potential shear band locations were then indirectly localized based on the positive–negative partitioning and abrupt amplitude changes in the predicted rotation angles. The results show that the DEM simulations agree well with the cumulative rotation-angle responses obtained from the laboratory tests, with mean absolute percentage errors of 9.79%, 6.03%, and 3.84% under the T1, T2, and T3 conditions, respectively. Within the shear band influence zone, the upper monitoring points mainly exhibit negative rotation-angle accumulation, whereas the lower monitoring points primarily show positive rotation-angle responses. A larger absolute rotation angle indicates a stronger controlling effect of the shear band on the corresponding monitoring point. The PCA + Gaussian model demonstrates strong overall performance in regional rotation-angle prediction, with MAE, RMSE, and CRPS values of 0.0803, 0.1024, and 0.0801, respectively. The model preserves the dominant deformation mode of the rotation-angle field and provides probabilistic prediction outputs. The proposed method enables the prediction of internal rotation-angle responses and facilitates the localization of potential shear band locations in gravelly soil slopes, providing data-driven technical support for precursor recognition and intelligent early warning of progressive slope failure.

## 1. Introduction

Slope instability and collapse are typical geological hazards in mountainous transportation and municipal engineering. Influenced by intense rainfall, toe excavation, and construction disturbances, slopes are prone to localized deformation and sliding failure [1]. Compared with homogeneous soil slopes, gravel–soil mixtures exhibit pronounced particle interlocking and meshing effects, and slope failure typically evolves through a progressive process involving shear band initiation, propagation, and eventual coalescence [2,3]. Therefore, identifying the location and evolution of internal shear bands prior to global failure is critical for precursor detection and early warning of gravel–soil slope instability.

The precise sensing of internal slope movement is closely linked to the accurate identification of shear band locations [4,5]. Monitoring approaches for internal slope deformation include borehole inclinometry, time-domain reflectometry (TDR) [6,7,8], global navigation satellite system (GNSS) [9,10,11], interferometric synthetic aperture radar (InSAR) [12,13,14,15], and crack monitoring techniques. These methods infer and locate internal deformation zones and potential slip surfaces by analyzing variations in inclination, electromagnetic wave reflection characteristics, surface displacement fields, and crack propagation patterns. However, they primarily rely on macroscopic displacement responses and exhibit limited capability in capturing localized internal deformation within the soil mass, making it difficult to effectively reveal the micromechanical evolution of shear band initiation and development.

In contrast, micro-electro-mechanical systems (MEMS) sensors, owing to their small size, ease of embedment, high sensitivity, and capability for real-time continuous monitoring, can capture localized deformation information within soil more finely, thereby addressing the limitations of conventional sensing technologies. Segalini et al. [16] developed an automated inclinometer monitoring system based on micro-electro-mechanical system technology and verified its applicability in landslide monitoring, demonstrating the potential of MEMS-based sensing techniques for capturing internal slope deformation characteristics. Subsequently, Wu et al. [17] proposed an MEMS-based method for internal displacement monitoring of slope soil, improving displacement inversion accuracy through time-domain integration of acceleration signals combined with error correction algorithms. Furthermore, Wu et al. [18] conducted model experiments on unstable highway slopes, demonstrating that embedded MEMS sensors can effectively track the evolution of internal soil displacement and maintain high sensitivity to localized responses prior to slope failure. Khan et al. [19] evaluated and calibrated various MEMS-IMU sensors, highlighting that sensor noise characteristics, attitude fusion algorithms, and gravity component separation methods are critical factors influencing the accuracy of real-time slope monitoring.

Although MEMS sensors have been demonstrated to be effective for monitoring internal slope displacement and attitude variations, two key challenges remain in shear band localization and progressive failure prediction. First, how can the correlation between MEMS monitoring responses and shear band initiation and evolution be systematically characterized? Existing studies primarily focus on displacement inversion or inclination variation patterns, while insufficient attention has been paid to rotational responses that can capture the relative motion characteristics across shear band interfaces. In fact, during shear band formation and propagation, soils on either side of the band typically exhibit counter-rotational deformation. The resulting distribution of positive and negative rotation zones, together with abrupt changes in rotation magnitude, can more directly indicate the location and extent of the influence of shear bands. Second, how can reliable predictive models that reflect shear band evolution be constructed using limited monitoring data? Due to constraints such as the high cost of physical model testing, the long-term requirements of field monitoring, and the scarcity of slope failure events, existing studies struggle to obtain sufficient datasets describing shear band evolution, leading to inadequate training of data-driven models and limiting the application of rotational response information in progressive failure identification and early warning. Therefore, there is an urgent need to develop a predictive framework that integrates experimental testing, numerical simulation, and data-driven methods to effectively extend internal rotational response datasets, reconstruct spatial rotation-angle fields, and facilitate the localization of potential shear bands in slopes.

In summary, to address the challenge of timely localization of shear bands during the progressive failure of gravel–soil slopes, this study proposes an MEMS-based shear band localization framework driven by rotational responses and machine learning prediction. First, scaled laboratory model tests are conducted, in which MEMS sensors are embedded in a stratified configuration within the slope to acquire time-series cumulative rotational responses under different gravel content conditions. Second, a discrete element method (DEM) numerical model is developed to investigate the spatial heterogeneity of the rotation-angle field during shear band formation and to provide supplementary validation of the experimental results. Finally, a data augmentation strategy combined with machine learning techniques is introduced to construct a regional rotation-angle field prediction model. Based on the predicted rotation-angle field, potential shear band initiation zones are subsequently localized according to the distribution of positive and negative rotation regions and the magnitude characteristics of the rotational responses. This study provides a new methodological framework for precursor identification, rotational-response-based shear band localization, and intelligent early warning of progressive failure in gravel–soil slopes.

## 2. Scale Model Test of Slope Collapse

### 2.1. Design of the Model Test

#### 2.1.1. Test Soil Samples

**Figure 1:**
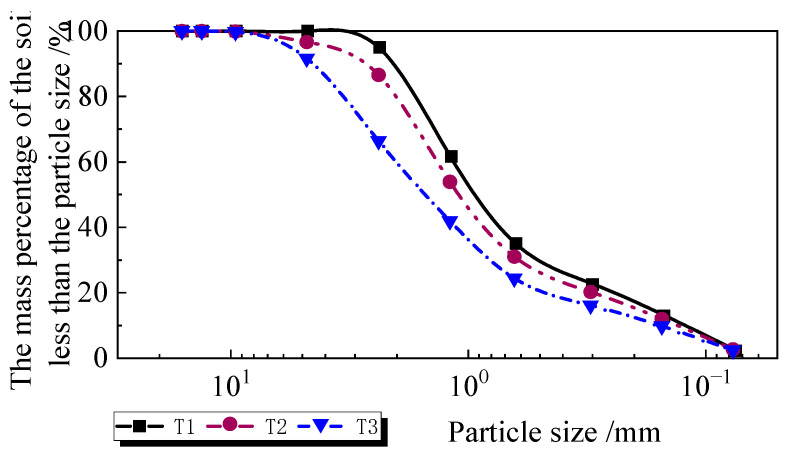
Particle Size Distribution Curves of the Test Groups.

The soil samples used in this experiment were collected from the Huajiang area of Lingchuan County, Guilin, Guangxi, representing typical local red clay characterized by a reddish-brown color, minor gravel content, a dense structure, and certain inherent structural strength. The samples were air-dried naturally and manually crushed using a wooden pestle before being passed through a 2 mm standard sieve to obtain purified red clay particles, which were then sealed to prevent variations in moisture content. The coarse sand and gravel were sieved and classified using 2.36 mm, 4.75 mm, 9.5 mm, and 13.2 mm standard sieves, dividing the gravel into four particle size ranges. Each fraction was weighed to quantify the mass proportion of each size group, providing a grading basis for constructing clumped particle structures in subsequent DEM simulations. According to the Code for Design of Building Foundation (GB 50007–2011) [20], the gravel–soil mixture was prepared by blending gravel from the 4.75–9.5 mm and 13.2–16 mm size ranges at a mass ratio of 1:1, while coarse sand (2.36–4.75 mm) was mixed with red clay at a 1:1 mass ratio. This ensured that the total mass fraction of particles larger than 2 mm exceeded 50%, satisfying the specification requirements for gravel soil. Based on this, three groups of laboratory slope model tests (T1–T3) were designed for comparison, with detailed soil–rock proportions and experimental parameters provided in Table 1, and the particle size distribution curves shown in Figure 1.

#### 2.1.2. Pilot Scheme

**Figure 2:**
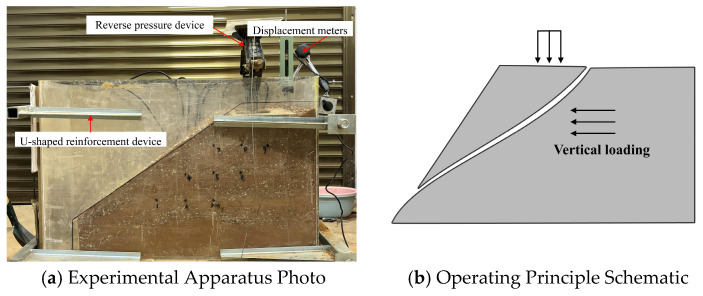
Experimental Apparatus and Schematic Illustration of the Loading Mechanism.

The model box has dimensions of 750 mm × 550 mm × 300 mm and is constructed using 8 mm thick tempered glass panels. To ensure structural integrity, four U-shaped reinforcement devices are installed around the perimeter of the model box to provide lateral restraint and reduce boundary deformation effects. The experimental setup and the operating principle of the loading system are shown in Figure 2.

Slope failure induced by surcharge loading at the crest is simulated using a reverse pressure device to apply controlled vertical displacement. The reverse pressure device is installed above the slope crest, and a steel loading plate measuring 200 mm × 150 mm × 3 mm is placed between the device and the slope surface to distribute the concentrated load uniformly. During the loading process, the upward movement of the reverse pressure device is restricted by the reaction constraint system, allowing the device to generate downward displacement and transfer the applied load through the loading plate to the slope crest.

The loading process is conducted at a constant displacement rate of 5 mm/min until the target displacement of 45 mm is reached. The applied vertical displacement induces progressive deformation within the slope, resulting in the initiation and propagation of shear bands. In addition, displacement meters are installed on the side walls of the model box to monitor possible out-of-plane deformation during loading. The monitoring results indicate that no significant lateral deformation occurs on the side walls, confirming that the boundary conditions remain stable and that the experimental system provides reliable loading conditions for investigating progressive slope failure.

The arrows indicate the direction of vertical loading applied to the slope model.

#### 2.1.3. MEMS Sensor System and Rotational Response Measurement

To monitor the particle rotational behavior during progressive failure of gravelly soil slopes, a WIT-HWT901B nine-axis MEMS sensor developed by WitMotion was employed in this study. The sensor is a high-performance attitude and heading reference system (AHRS), which integrates a three-axis accelerometer, a three-axis gyroscope, and a three-axis magnetometer. The accelerometer measures the acceleration response, including gravity components; the gyroscope captures angular velocity variations; and the magnetometer provides geomagnetic information for attitude correction. The fusion of multi-source inertial measurements enables continuous monitoring of the internal rotational response of soil particles during shear deformation.

The sensor has a compact structure with dimensions of 55.0 mm × 36.8 mm × 23.0 mm, which minimizes disturbance to the soil structure during embedding. The attitude measurement accuracy reaches 0.05° in the X–Y directions and 1° in the Z direction. The sensor supports TTL232 serial communication with a maximum output frequency of 200 Hz. Considering the duration of the model test and the balance between temporal resolution and data storage capacity, the sampling frequency was set to 20 Hz in this study. The operating voltage ranges from 9.0 to 36.0 V, and the power consumption is lower than 40 mA.

The rotational response was obtained through the embedded attitude calculation algorithm of the AHRS. The raw acceleration, angular velocity, and geomagnetic signals were synchronously collected and fused using a Kalman-filter-based attitude estimation algorithm. After coordinate transformation and drift correction, the attitude variation at each monitoring point was converted into cumulative rotation-angle responses. The positive and negative signs of the rotation angle were defined according to the unified sensor coordinate system, ensuring the consistency of rotational response comparisons among different monitoring points.

**Figure 3:**
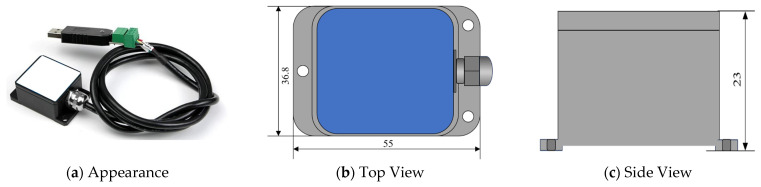
HWT901B Sensor Size Map (Unit: mm).

During the experiment, multiple MEMS sensors were embedded at different depths and horizontal positions within the slope model. The sensor arrangement was designed according to the potential development region of the shear band. Monitoring points were distributed above and below the potential shear zone to capture the differential rotational responses associated with shear localization. Figure 3 presents the physical appearance, structural dimensions, and installation configuration of the MEMS sensor.

All MEMS sensors were installed with the same orientation before testing to eliminate the influence of coordinate inconsistency. As illustrated in Figure 3, the X-axis of the sensor was aligned with the longitudinal direction of the slope, the Y-axis was perpendicular to the slope surface, and the Z-axis was vertically downward. The rotational response around the Z-axis was selected as the primary indicator because it is sensitive to the relative particle rotation induced by shear localization. According to the defined coordinate system, clockwise rotation was assigned a positive value, whereas counterclockwise rotation was assigned a negative value.

### 2.2. DEM Numerical Simulation

#### 2.2.1. Establishment of the Simulation Model

To obtain the macroscopic physical and mechanical parameters of the experimental soil required for DEM simulations [21,22], direct shear tests were conducted using a ZFY-1A strain-controlled four-unit direct shear apparatus under different normal stress conditions. The shear stress–displacement responses of the gravelly soil samples were measured during the tests, and the shear strength parameters were determined based on the Mohr–Coulomb failure criterion. The macroscopic parameters of the tested soil are presented in Table 2.

The parameters listed in Table 2 represent the intrinsic mechanical properties of the tested gravelly soil samples obtained from direct shear tests rather than artificially assigned parameters. Specifically, *φ* represents the internal friction angle obtained from the slope of the shear strength envelope, *c*_app_ represents the apparent cohesion obtained from the intercept of the fitted shear strength envelope, and μ represents the equivalent macroscopic friction coefficient derived from the internal friction angle. These parameters were subsequently used as the basis for DEM model calibration under different gravel content conditions.

The shear strength relationship of the gravelly soil can be expressed as:

(1) $$\tau =c_{app}+\sigma_{n}tan\varphi$$

where τ represents the shear strength, σn represents the normal stress, φ represents the internal friction angle, and capp represents the apparent cohesion induced by particle interlocking, particle rearrangement, and passive resistance effects during shearing.

The stress–strain relationship obtained from the direct shear tests exhibited typical strain-hardening characteristics. With increasing shear strain, the shear stress gradually increased and finally approached a relatively stable value, which was considered the representative shear strength for parameter determination. Meanwhile, the non-zero intercept of the shear stress–normal stress relationship indicated the existence of apparent cohesion. According to the passive resistance theory proposed by Kang et al. [23], the apparent cohesion does not represent intrinsic mineral cohesion but results from particle interlocking, meshing, and rearrangement effects. During shearing, relative movement between particles along rough contact surfaces induces continuous adjustment of particle positions and orientations, generating additional resistance that is macroscopically reflected as *c*_app_.

In addition to the macroscopic mechanical parameters obtained from direct shear tests, microscopic contact parameters required for the rolling resistance linear contact model were also defined. Unlike the macroscopic parameters, these microscopic parameters cannot be directly measured using conventional laboratory instruments. Therefore, the microscopic contact parameters were determined through DEM calibration by comparing the simulated mechanical responses with the experimental results and referring to previous studies. These parameters characterize the inter-particle interaction behavior during DEM simulations, including contact modulus, particle density, particle friction coefficient, contact stiffness ratio, and damping coefficient. The microscopic parameters adopted in the DEM model are summarized in Table 3.

The macroscopic parameters and microscopic contact parameters play different roles in controlling the simulated mechanical response. The macroscopic parameters, including φ and *c*_app_, mainly determine the shear strength and deformation resistance of the gravelly soil, whereas the microscopic parameters regulate particle-scale interactions, including sliding, rearrangement, and energy dissipation. Therefore, appropriate calibration of these parameters is essential for reproducing the shear band evolution and rotational response characteristics observed in the laboratory tests.

**Figure 4:**
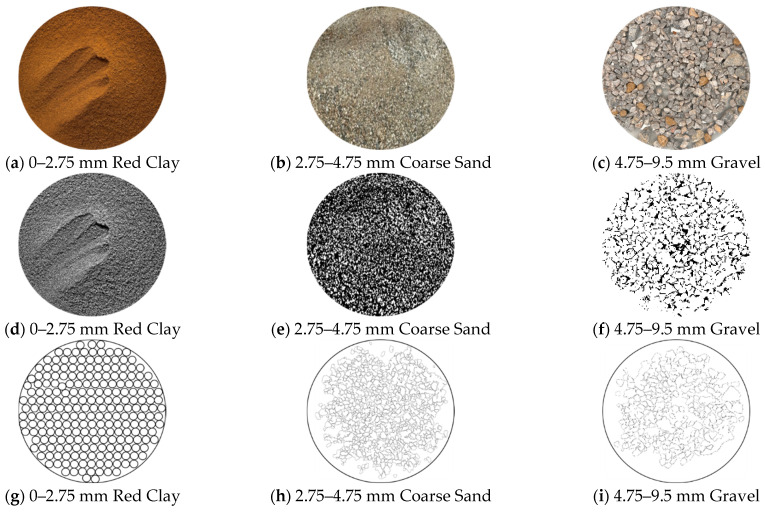
Particle Contour Images after Grayscale Processing and Binarization in FIJI.

Coarse sand and gravel particles were subjected to two-dimensional image acquisition. Geometric correction was performed in Photoshop to obtain standardized images with a resolution of 1960 × 1960 pixels. Subsequently, grayscale conversion and binarization were carried out in FIJI to generate TIFF-format contour images. Red clay particles were simplified as circular shapes, while the characteristic contours of the remaining particles were extracted using AutoCAD 2020 (Autodesk Inc., San Francisco, CA, USA) to construct a particle geometry database, as shown in Figure 4.

**Figure 5:**
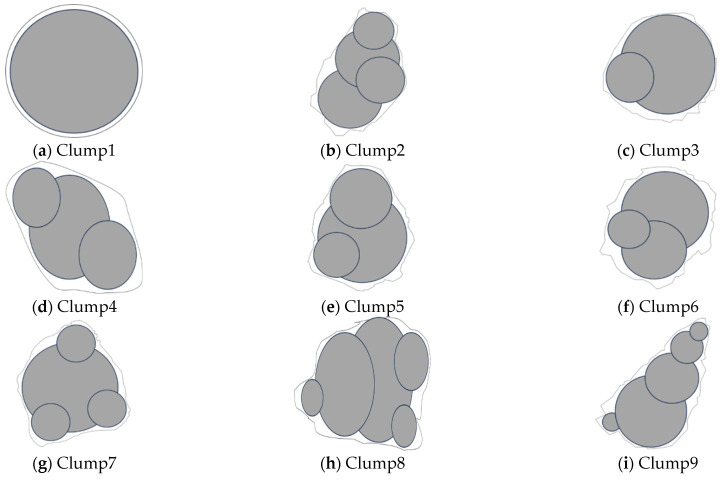
Clump Models of Particle Clusters.

Nine representative particle contour images were imported into PFC2D 5.0 (Particle Flow Code in Two Dimensions, Itasca Consulting Group, Inc., Minneapolis, MN, USA), where the Clump function (Pebble packing method) was employed to generate irregular clump particle models. The key parameters include the surface smoothing distance (distance), set to 150 for red clay, 130 for coarse sand, and 110 for gravel, and the particle size ratio (ratio), ranging from 0 to 1, which was selected as a trade-off between model accuracy and computational efficiency. Ultimately, nine representative Clump particle models were generated, as shown in Figure 5.

**Figure 6:**
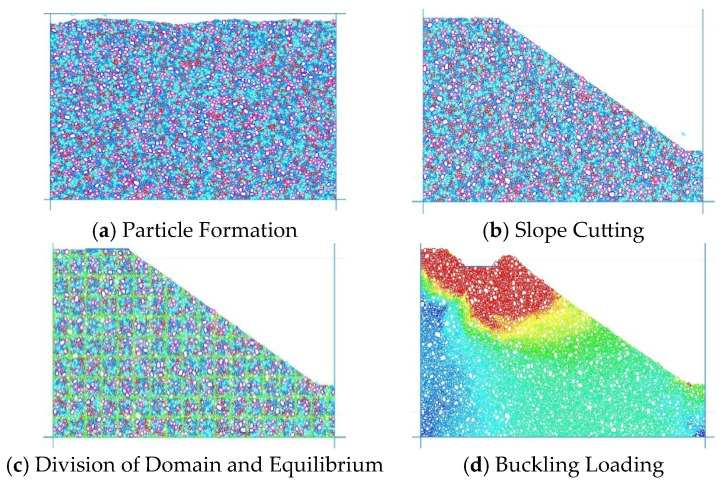
Three Groups of Numerical Simulations of the Whole Process of Slope Failure.

A rigid boundary wall was established, within which a particle assembly with a Gaussian particle size distribution was generated. Gravitational loading was applied, followed by mechanical equilibrium calculations to bring the model to an initial stress-balanced state. Subsequently, the designed slope geometry was created by cutting along the predefined slope profile, removing particles outside the boundary domain, and performing a second equilibrium calculation to eliminate residual stresses. Observation lines were arranged within the model at 40 mm intervals in both the horizontal and vertical directions to form a measurement grid. A loading plate with a length of 100 mm was installed at the slope crest, and a constant downward displacement was applied at a rate of 5 mm/min until the vertical settlement reached 45 mm, at which point the loading was terminated, as shown in Figure 6. In the DEM visualization, different colors were used to distinguish individual particles in the assembly, while the color distribution in the loading stage represents the spatial variation of the simulated response.

#### 2.2.2. Model Validation

**Figure 7:**
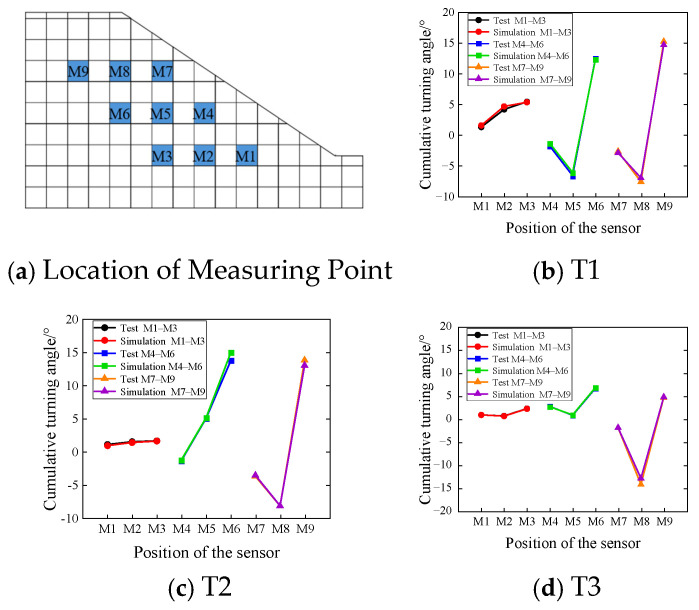
Comparison of Test–Simulation Cumulative Turning Angles for the Three Groups.

To validate the rationality of the DEM model and the rolling resistance linear contact parameters, the numerical simulation domain was discretized into a 16 × 9 grid, as shown in Figure 7a. The experimental results from the T1, T2, and T3 laboratory slope models were compared with the corresponding DEM simulation results. The cumulative rotation angle at each monitoring point was used as the evaluation metric, and the mean absolute percentage error (MAPE) between the experimental and simulated values was calculated, as shown in Figure 7. The MAPE values for the T1, T2, and T3 cases were 9.79%, 6.03%, and 3.84%, respectively, all indicating a relatively low level of discrepancy and demonstrating that the simulation results obtained from the developed DEM model can effectively reproduce the internal rotational response characteristics of the slope. It is observed that the deviation between the experimental and simulated results decreases with increasing gravel content. This is mainly attributed to the enhanced particle interlocking and skeletal support provided by the higher gravel content, which promotes the localization of shear deformation into more confined regions, thereby making the shear band location and dominant deformation zone more distinct. Under such conditions, the DEM simulation provides more stable reproduction of rotational responses and deformation localization processes. Overall, the rolling resistance linear contact model established in this study can effectively reproduce the rotational response characteristics during shear band formation in gravel–soil slopes under different gravel contents, providing a reliable numerical basis for subsequent rotation field analysis and shear band localization.

### 2.3. Analysis of Shear Band Rotation Response Characteristics

#### 2.3.1. Relationship Between Rotation Data and Shear Band Position

The results of the laboratory model tests indicate that the internal rotational responses of the slope exhibit a clear correspondence with the formation and propagation of shear bands. Within the influence zone of the shear band, monitoring points in the upper region predominantly show cumulative negative rotation, whereas those in the lower region are characterized by positive rotational responses, forming a stable rotational partition between the upper and lower blocks. This phenomenon suggests that, during relative sliding along the shear band, the soil on both sides undergoes oppositely directed rotational deformation. In addition, larger absolute rotation magnitudes indicate a stronger control exerted by the shear band on the corresponding measurement points. Therefore, the spatial distribution of positive and negative rotation zones, together with abrupt changes in rotation magnitude, can serve as important indicators for identifying potential shear band locations.

**Figure 8:**
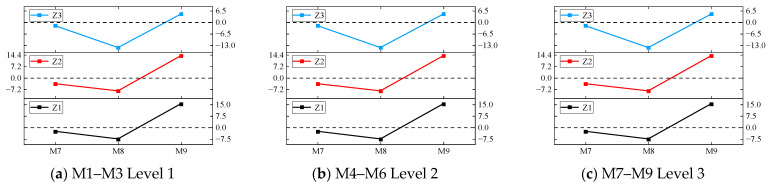
Sensor Rotation Data from the Indoor Model Tests (Unit: rad).

Monitoring points M1–M3 in the first layer are located near the bottom of the model box and are strongly influenced by boundary constraints. Their rotational responses primarily reflect the overall coordinated deformation behavior of the lower part of the slope. As the gravel content increases, the cumulative rotation angle in this layer shows an overall decreasing trend, as illustrated in Figure 8a. Under the T1 condition, the cumulative rotations at M1, M2, and M3 are 1.24°, 4.13°, and 5.38°, respectively. Under the T2 condition, these values decrease to 1.06°, 1.49°, and 1.62°, and further change to 0.89°, 0.68°, and 2.28° under the T3 condition. These results indicate that, with the strengthening of the gravel skeleton effect, the extent of shear deformation diffusion toward the slope base is reduced, and the internal deformation gradually transitions from a globally distributed pattern to a more localized concentration.

Monitoring points M4–M6 in the second layer are located in the middle of the slope and represent a highly sensitive zone for shear band initiation and spatial migration. As shown in Figure 8b, under the T1 condition, M4 and M5 exhibit negative rotations of −1.86° and −6.70°, respectively, while M6 reaches a positive rotation of 12.47°, resulting in a within-layer positive–negative rotation difference of 19.17°. This indicates that the layer is strongly controlled by the shear band and exhibits oppositely directed rotational responses. Under the T2 condition, the rotation at M5 changes from negative to positive (5.03°), while M6 increases to 13.75°, suggesting that the shear band crossing position migrates from between M5 and M6 to between M4 and M5. Under the T3 condition, M4 and M5 shift to positive rotations of 2.77° and 0.84°, respectively, while M6 decreases to 6.68°, indicating that, under high gravel content conditions, the extent of mid-slope shear deformation further contracts and the dominant shear band zone continues to migrate upward. These results demonstrate that the rotational responses in the second layer can sensitively capture both the spatial evolution and the controlling intensity of the shear band.

Monitoring points M7–M9 in the third layer are located near the slope crest and exhibit the most pronounced responses to upper sliding mass movement and shear band penetration. As shown in Figure 8c, under the T1 condition, M7 and M8 show negative rotations of −2.73° and −7.69°, respectively, while M9 reaches a positive rotation of 15.23°, indicating a clear within-layer differentiation of positive and negative rotational responses and suggesting that the shear band primarily passes between M8 and M9. Under the T2 condition, the negative rotations at M7 and M8 increase to −3.74° and −8.23°, respectively, while M9 remains at 13.82°, indicating that the shear band still crosses between M8 and M9, but the rotational responses become further concentrated in this key region. Under the T3 condition, the absolute rotation at M8 increases to 14.24°, making it the dominant response point, while M7 and M9 exhibit rotations of −1.88° and 4.67°, respectively. This suggests that, under high gravel content conditions, deformation localization in the crest region is enhanced, and the dominant shear band pathway becomes more clearly defined.

A comprehensive analysis of the monitoring results across different layers indicates that increasing gravel content significantly alters the spatial distribution of shear deformation within the slope. Under low gravel content conditions, shear deformation exhibits a relatively wide diffusion pattern. In contrast, under high gravel content conditions, the enhanced interlocking and skeletal support of gravel particles promote the progressive concentration of shear deformation toward a limited number of key monitoring points. The spatial partitioning of positive and negative rotations, abrupt changes in rotational responses among points within the same layer, and zones of high absolute rotation collectively constitute macroscopic criteria for shear band localization. These observations provide an experimental basis for subsequent shear band identification using region-based rotational prediction methods.

#### 2.3.2. Relationship Between Rotation Amplitude and Shear Band Distance

**Figure 9:**
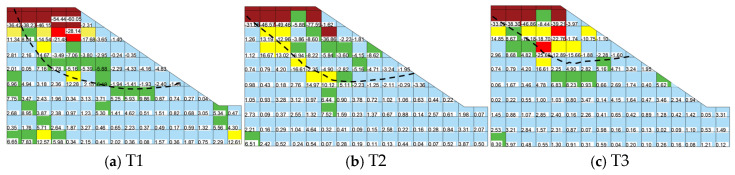
Three Groups of Simulated Cumulative Rotation-Angle Nephograms. The color scale represents the magnitude of cumulative rotation angle, with blue, green, yellow, and red corresponding to gradually increasing rotation-angle responses.

Figure 9 presents the cumulative rotation contour maps obtained from DEM simulations under the T1–T3 conditions. Overall, the internal rotational field of the slope exhibits pronounced spatial heterogeneity, with high-rotation zones primarily concentrated beneath the loading area at the slope crest and in the vicinity of potential shear band regions, while low-rotation responses dominate the bedrock and areas far from the shear band. The sign of the rotation reflects differences in the local particle rotation direction, whereas the absolute magnitude of rotation characterizes the intensity of particle rolling, interlocking, and fabric rearrangement. Therefore, regions with high absolute rotation values can serve as important indicators of shear band activity and its spatial evolution.

Under the T1 condition, high-rotation zones are primarily distributed near the slope crest and within a shallow banded region beneath it, with local rotational magnitudes reaching 40–60°. Within these highly deformed zones, alternating positive and negative rotation patterns are also observed. This indicates that, under low gravel content conditions, the upper slope exhibits pronounced bending–rotation and tensile separation tendencies. The shear band develops as a relatively continuous banded structure in space and progressively propagates along the potential slip surface toward full connectivity. Outside this highly deformed zone, rotational responses in the mid- and lower-slope regions are relatively weak, suggesting limited disturbance of the underlying bedrock. Overall, slope deformation is dominated by a combination of coordinated bulk movement and localized rotational deformation.

Under the T2 condition, pronounced high-rotation responses are still observed near the slope crest, with a peak value reaching 77.59°. In contrast to the relatively continuous banded distribution observed under the T1 condition, the high-rotation zones in the T2 case exhibit a more patchy and discrete pattern, with multiple intermittently distributed clusters of high rotation forming along the shear band path. This indicates that the incorporation of gravel particles alters the homogeneity of the internal contact network of the soil, making shear deformation more strongly governed by localized particle interlocking, rolling, and rearrangement processes. Although the dominant shear band pathway remains identifiable, its continuity is partially disrupted and exhibits slight deviations. Meanwhile, rotational effects within the shear band are enhanced, leading to a higher degree of deformation localization.

Under the T3 condition, the high-rotation zone no longer forms a single, smooth, and continuous banded structure but instead exhibits more pronounced undulations, segmentation, and non-planar characteristics. Multiple medium-to-high rotation units are observed in the crest and upper–mid-slope regions, including concentrated zones with positive rotations on the order of 40° as well as clustered regions with negative rotations of approximately 20–30°. Compared with the T1 and T2 conditions, the spatial distribution of high absolute rotation areas in the T3 case is more irregular and covers a broader extent, indicating that the shear band evolves from a thin sliding surface into a localized failure zone with finite thickness. This behavior can be attributed to the increased gravel content, where enhanced interlocking of the gravel skeleton suppresses the formation of smooth slip surfaces. As a result, shear deformation must be accommodated through particle rolling, interlocking-induced bypassing, and local rearrangement, leading to a rougher shear band geometry, more diffuse boundaries, and pronounced heterogeneous shear characteristics.

A comparative analysis of the three cases indicates that, with increasing gravel content, the rotational response of the shear band gradually evolves from a relatively continuous banded concentration to a more patchy, undulating, and thickened distribution governed by the particle skeleton. Under high gravel content conditions, shear deformation must be accommodated within a more complex particle contact network through mechanisms such as rolling, interlocking, and structural rearrangement, resulting in enhanced heterogeneity of the shear band and increased energy dissipation characteristics. This evolutionary trend is consistent with the macroscopic observations from the direct shear tests, where the T3 condition exhibits increased shear band roughness and a transition toward a thicker failure zone.

## 3. Prediction of Shear Band Position Based on Machine Learning

### 3.1. MEMS Data Augmentation Method Based on Probability Density Estimation

#### 3.1.1. Methodology

To address the limited sample size, strong nonlinearity, pronounced stochastic disturbances, and difficulty in characterizing multimodal distributions of MEMS rotational time-series data, this study develops a Probability Density Layer–Generative Adversarial Network (PDL-GAN) data augmentation framework. First, a Probability Density Layer (PDL) is introduced to perform non-parametric kernel-based density estimation of MEMS time-series features, extracting low-dimensional prior representations that characterize the underlying distribution of real samples. Subsequently, the obtained prior features are incorporated into a Generative Adversarial Network (GAN), where the generator and discriminator are trained in an adversarial manner to synthesize augmented samples that are consistent with the statistical and dynamic characteristics of real rotational responses.

In the data preprocessing stage, a sliding window approach is employed to segment the MEMS rotational time-series signals. Let the window length be (L) and the step size be (S). Within each time window, statistical features including the peak value, root mean square (RMS), standard deviation, and rotational increment are extracted to construct the feature space of real samples. A larger window length helps mitigate the influence of random noise, while a smaller step size increases sample overlap and temporal continuity, thereby enhancing the model’s capability to represent the evolution process of rotational responses.

The Probability Density Layer (PDL) is designed to learn the statistical distribution characteristics of MEMS features derived from both experimental measurements and DEM simulations. Its fundamental idea is to map the original feature tensors into a high-dimensional distribution space, where nonlinear transformations and tensor compression are applied to obtain a probabilistic density representation. After normalization, the probability density function is further transformed into a probability mass function to characterize the dominant distribution patterns of real samples in the latent space. This process does not rely on any predefined distributional assumptions, enabling it to better accommodate the non-Gaussian, multimodal, and stochastic fluctuation characteristics inherent in MEMS data.

In the sample generation stage, the low-dimensional probabilistic features extracted by the PDL are used as prior inputs to the generator (G) and are jointly mapped with a random noise variable (z) into the MEMS feature space to produce synthetic rotational feature samples (G(z)). The discriminator (D) is employed to distinguish whether the input samples originate from the real dataset or are generated by the generator. The training objective of the GAN can be expressed as:

(2) $$L\left(D,G\right)=Ex\sim p_{data}\left(x\right)\left[\log D\left(x\right)\right]+Ez\sim p\left(z\right)\left[\log\left(1-D\left(G\left(z\right)\right)\right)\right]$$

Through alternating optimization between the generator and the discriminator, the generated samples gradually approximate the true distribution of MEMS rotational features. When the discriminator can no longer effectively distinguish between real and synthetic samples, the model reaches a dynamic equilibrium, and a well-trained generator is obtained for data augmentation purposes.

To ensure the validity of the generated samples, the Bray–Curtis distance is introduced to evaluate and filter the distributional consistency between generated and real samples. Its expression is given as follows:

(3) $$d_{B-C}(r,g)=\frac{\sum_{i=1}^{n}\left|r_{i}-g_{i}\right|}{\sum_{i=1}^{n}\left(r_{i}+g_{i}\right)}$$

In this expression, $d_{B-C}$ denotes the Bray–Curtis distance between the real sample r and the generated sample g; n represents the feature dimensionality (with *n* = 2 in this study); $r_{i}$ and $g_{i}$ correspond to the i-th components of the real and generated two-dimensional feature vectors, respectively. This metric is embedded into a rejection sampling procedure: when the Bray–Curtis distance between a generated sample and a real sample is below a predefined threshold, the generated sample is considered to exhibit satisfactory statistical consistency with the real MEMS responses and is retained; otherwise, it is treated as an anomalous synthetic sample and discarded. Through this procedure, an augmented dataset with statistical consistency, temporal continuity, and physical plausibility is ultimately constructed, providing a reliable data foundation for subsequent regional rotation prediction and shear band localization.

#### 3.1.2. Data Enhancement Quality Analysis

**Figure 10:**
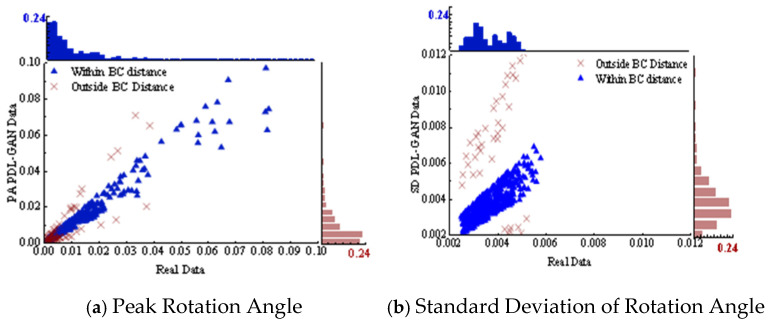
B–C Distance Discrimination Between Generated and Real Samples.

To validate the effectiveness of the proposed PDL-GAN data augmentation method, MEMS rotational response feature parameters were selected as evaluation objects, with peak rotation and the standard deviation of rotation adopted as key indicators. The Bray–Curtis (B–C) distance was used to assess the distributional consistency between generated and real samples in the feature space. Figure 10 presents the B–C distance-based discrimination results between PDL-GAN-generated samples and real samples, where blue triangles denote valid generated samples satisfying the B–C distance threshold constraint, and red crosses represent anomalous generated samples exceeding the threshold. It can be observed that the valid samples constrained by the B–C distance are primarily distributed within the dominant feature domain of the real samples and exhibit strong consistency trends across both the peak rotation and the standard deviation of rotation dimensions. In contrast, samples exceeding the B–C threshold deviate significantly from the main distribution of the real data and show pronounced dispersion. These results demonstrate that the B–C distance can effectively identify and remove generated samples that deviate from the statistical characteristics of the real data, thereby improving the distributional consistency and reliability of the augmented dataset. This confirms that the PDL-GAN model not only expands the sample size of MEMS rotational responses but also preserves the consistency of peak response and fluctuation characteristics between generated and real data. The augmented dataset filtered by the B–C distance exhibits strong statistical validity, providing a reliable data foundation for subsequent regional rotation prediction and shear band localization models.

### 3.2. Predictive Tasks and Model Settings for Machine Learning

#### 3.2.1. Machine Learning Model Training

A block-wise time-ordered partitioning strategy was adopted to divide the original time-series data into training, validation, and test sets, rather than randomly shuffling sliding-window samples. This strategy prevents adjacent windows derived from the same original sequence from simultaneously appearing in both the training and test sets, thereby better aligning with engineering application scenarios where future rotational responses are predicted based on historical monitoring data. In the dataset split, the training, validation, and test sets account for 70%, 15%, and 15% of the data, respectively, corresponding to 560, 120, and 120 samples. The training set is used for model parameter learning, the validation set is used for hyperparameter tuning and early stopping control, and the test set is reserved exclusively for final performance evaluation. It should be noted that the generated samples proposed in Section 3.1 are used only to augment the training set and are not included in the validation or test sets, ensuring that model evaluation is conducted solely based on real experimental samples and DEM simulation data.

Five machine learning models are selected for comparison: Baseline, PCA + Gaussian, XGBoost, VAR, and Transformer. The Baseline model serves as a reference benchmark to provide reproducible performance standards. PCA + Gaussian is used to extract dominant the low-dimensional evolutionary modes of the rotational field and output probabilistic predictions. XGBoost is employed to capture nonlinear mappings among rotational features. VAR is used to characterize multivariate linear temporal dependencies. Transformer was adopted to model long-range dependencies in rotational sequences. All models are evaluated under consistent data partitioning schemes, input–output formats, and performance metrics to ensure fair and unbiased comparison.

#### 3.2.2. Model Parameter Settings

To ensure fair comparison among different models, all input features are standardized using statistics computed from the training set, while the validation and test sets are transformed using the same normalization parameters. After training, all models produce future rotational angle predictions on the same test set, which are further used for shear band potential location identification. The main parameter settings of each model are summarized in Table 4.

The Baseline model adopts a regression framework with L2 regularization, serving as a performance benchmark for the regional rotation prediction task. The PCA + Gaussian model first applies principal component analysis (PCA) to extract dominant low-dimensional evolutionary modes of the rotational field, with the number of principal components determined based on cumulative variance contribution and validation performance, and fixed at five. A Gaussian probabilistic model is then used to output both the predicted mean and the 95% confidence interval of future rotational angles.

The XGBoost model is employed to capture nonlinear mappings among multi-regional rotational responses, with model complexity controlled via early stopping on the validation set. The VAE model adopts a lightweight encoder–decoder architecture to evaluate the effectiveness of generative representation learning in small-sample rotational prediction tasks. The Transformer model uses a lightweight attention-based architecture to assess the effectiveness of long-range dependency modeling for future rotational angle prediction.

### 3.3. Analysis of Forecast Results

#### 3.3.1. Evaluation Indicators

To evaluate the applicability of different machine learning models for long-term regional rotational angle prediction, this study establishes a comprehensive evaluation framework from two perspectives: point prediction error and probabilistic prediction quality. Point prediction errors are quantified using the mean absolute error (MAE) and root mean square error (RMSE), where MAE measures the average deviation between predicted and observed values, while RMSE is more sensitive to large errors during abrupt change phases and reflects the robustness of the models under non-stationary rotational responses.

Probabilistic prediction quality is assessed using the continuous ranked probability score (CRPS), 95% prediction interval coverage (Coverage95), and interval width, which respectively measure the consistency between predicted distributions and observations, the ability of prediction intervals to capture true values, and the sharpness of uncertainty intervals. The evaluation results for each model are presented in Table 3.

In this study, historical rotational angle sequences consisting of 1000 sampling points are used as inputs to predict the future rotational responses over the subsequent 100 sampling points. Compared with one-step-ahead prediction, this task imposes higher requirements on the model’s capability for long-term trend extrapolation, temporal dependency representation, and uncertainty quantification. Therefore, the evaluation of the models not only focuses on the magnitude of the prediction errors, but also considers whether the predicted intervals can adequately cover the true values while maintaining a reasonable interval width.

MAE is defined as:

(4) $$MAE=\frac{1}{N}\sum_{I=1}^{N}\left|y_{i}-\hat{y_{i}}\right|$$

RMSE is defined as:

(5) $$RMSE=\sqrt{\frac{1}{N}\sum_{I=1}^{N}\left|y_{i}-\hat{y_{i}}\right|}$$

where y_i_ denotes the true value, $\hat{y_{i}}$ denotes the predicted value, and N is the number of samples. Among these metrics, MAE better reflects the overall average deviation, whereas RMSE is more sensitive to a small number of large errors and thus more effectively captures the model’s robustness during abrupt change stages.

For probabilistic prediction, this study employs the Continuous Ranked Probability Score (CRPS) to quantify the consistency between the predicted distribution and the observed values. It is defined as:

(6) $$CRPS\left(F,y\right)={\int_{-\infty }^{+\infty }\left(F\left(x\right)-1\left(x\geq y\right)\right)}^{2}dx$$

where (F(x)) denotes the cumulative distribution function (CDF) of the predicted distribution, and (y) represents the observed value. Coverage95 denotes the proportion of true values falling within the 95% prediction interval, while Interval Width represents the average width of the corresponding prediction interval. An optimal probabilistic prediction model should achieve adequate Coverage95 while maintaining a lower CRPS and a narrower Interval Width, thereby balancing predictive reliability and interval discriminative capability.

#### 3.3.2. Prediction Accuracy Analysis

Table 5 presents a comparison of regional rotational angle prediction performance among different machine learning models. It can be observed that significant performance differences exist across the models in this task. The Baseline and PCA + Gaussian models achieve the lowest point prediction errors, with MAE values of 0.0801 and 0.0803, and RMSE values of 0.1004 and 0.1024, respectively. The Baseline model slightly outperforms PCA + Gaussian, with reductions of 0.0002 in MAE and 0.0020 in RMSE, indicating a marginal advantage in point prediction accuracy. This suggests that MEMS rotational sequences exhibit strong temporal continuity, allowing even simple benchmark models to effectively preserve the dominant evolutionary trends of future rotations.

Compared with XGBoost, PCA + Gaussian reduces MAE from 0.0926 to 0.0803, corresponding to a 13.28% decrease, and reduces the RMSE from 0.1302 to 0.1024, corresponding to a 21.35% decrease. This indicates that PCA effectively extracts dominant deformation modes from multi-point rotational responses, reducing redundancy and suppressing local noise, while the Gaussian model enables stable extrapolation in the reduced-dimensional feature space, thereby improving long-term prediction performance.

The VAR and Transformer models exhibit significantly higher prediction errors than the other methods. Compared with PCA + Gaussian, VAR increases the MAE and RMSE by 80.95% and 95.70%, respectively, while the Transformer increases them by 133.75% and 150.59%. This indicates that linear autoregressive models are unable to capture the nonlinear and non-stationary characteristics of rotational responses, whereas more complex deep learning models are adversely affected by noise disturbances and local fluctuations under the current sample size, failing to achieve superior predictive performance.

From the perspective of probabilistic prediction metrics, both PCA + Gaussian and the Baseline model achieve identical CRPS values of 0.0801, which are lower than those of XGBoost, VAR, and the Transformer, indicating higher consistency between the predicted distributions and the observed data. The Coverage95 values of all models exceed 0.95; however, although the Transformer achieves the highest Coverage95 (0.9966), it also exhibits the highest MAE, RMSE, and CRPS, demonstrating that coverage alone cannot serve as a sufficient criterion for model performance evaluation.

The Interval Width of PCA + Gaussian is 3.7883, which is close to that of the Baseline model (3.7885), while simultaneously maintaining the lowest CRPS. This indicates that PCA + Gaussian achieves a favorable balance between distributional consistency and uncertainty control.

In summary, the Baseline model achieves the best performance in terms of point prediction errors and provides an effective benchmark for evaluating regional rotational response prediction. However, the prediction objective of this study is not limited to minimizing errors at individual monitoring points but aims to reconstruct the spatial evolution characteristics of the regional rotation-angle field and further identify potential shear band locations.

Although PCA + Gaussian exhibits slightly higher MAE and RMSE values than the Baseline model, it provides additional advantages in extracting dominant deformation modes and preserving spatial correlations among multiple monitoring points. Through PCA decomposition, the complex rotational responses are transformed into several dominant deformation patterns associated with shear evolution, while the Gaussian probabilistic model enables uncertainty-aware regional field reconstruction. Therefore, the predicted rotation-angle distribution maintains better spatial continuity and physical interpretability, which are essential for identifying shear bands based on rotational partitioning characteristics and abnormal amplitude variations.

Considering that shear band localization relies on regional deformation patterns rather than isolated point predictions, PCA + Gaussian was selected as the primary prediction framework in this study.

#### 3.3.3. Location Analysis of the Shear Band

**Figure 11:**
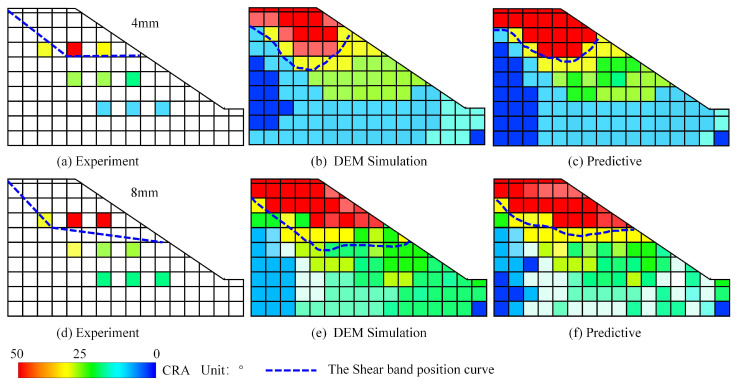
Comparison of Shear Band Localization Results Using Different Methods.

Figure 11 compares the shear band localization results obtained by different methods. Overall, all three results exhibit a consistent spatial pattern characterized by high rotational concentration in the upper slope and low rotational distribution in the lower region, indicating that the rotational field can effectively capture zones of concentrated shear deformation within the slope. At the 4 mm loading stage, the PCA + Gaussian predictions successfully capture the high-rotation zone beneath the slope crest, and the predicted shear band location is generally consistent with the experimental observations in terms of the overall trend, demonstrating the model’s capability to identify the early-stage development of the shear band.

When the displacement increases to 8 mm, the high-rotation zone further propagates into the interior of the slope. The PCA + Gaussian results continue to reflect the evolution from localized initiation to internal development of the shear band, and the predicted shear band trajectory remains in good agreement with the experimental results. Compared with DEM simulations, the PCA + Gaussian model does not require complex particle contact computations and can rapidly output regional rotational fields and shear band locations after training. This indicates that the proposed model achieves favorable predictive performance while significantly improving computational efficiency for shear band localization, highlighting the advantages of machine learning approaches in the rapid identification of progressive slope failure.

## 4. Conclusions

To address the challenge of timely identification of internal shear band locations during the progressive failure of gravel–soil slopes, this study integrates laboratory-scale model tests, DEM numerical simulations, PDL-GAN-based data augmentation, and machine learning prediction methods to investigate the relationship between internal rotational responses and shear band evolution under different gravel contents. A regional rotation prediction-based method for identifying potential shear band locations is proposed. The main conclusions are summarized as follows:

(1) A coupled analytical framework integrating MEMS rotational angle monitoring and DEM numerical simulation is established for shear band identification in gravel–soil slopes. Laboratory-scale model tests are conducted to obtain cumulative rotational responses within the slope under three different gravel content conditions (T1, T2, and T3). In the DEM simulations, the geometric morphology of gravel particles is represented through particle image reconstruction and Clump-based particle agglomeration, and the slope failure process is simulated using a rolling resistance linear contact model. The mean absolute percentage errors between the experimental and simulated cumulative rotations are 9.79%, 6.03%, and 3.84% for the T1, T2, and T3 cases, respectively, demonstrating that the proposed DEM model can effectively reproduce the rotational response characteristics associated with shear band formation in gravel–soil slopes.

(2) The spatial heterogeneity of MEMS rotational responses during shear band formation and the influence mechanism of gravel content are elucidated. Within the shear band influence zone, upper monitoring points predominantly exhibit cumulative negative rotations, whereas lower points show positive rotational responses, forming an oppositely directed rotational deformation pattern between the upper and lower blocks. With increasing gravel content, the cumulative rotation at the first monitoring layer decreases overall, indicating a reduced extent of shear deformation diffusion toward the slope base. In contrast, the second and third monitoring layers are more sensitive to shear band initiation, migration, and propagation processes.

Under low gravel content conditions, the rotational response associated with the shear band exhibits a relatively continuous banded distribution. Under high gravel content conditions, due to the enhanced effects of the particle interlocking, rolling, and local rearrangement of gravel particles, high-rotation zones gradually evolve into a more patchy, undulating, and locally thickened pattern. Consequently, the shear band transitions from a thin slip surface to a heterogeneous localized failure zone with finite thickness. Therefore, the spatial partitioning of positive and negative rotations, abrupt rotational changes within the same layer, and regions with high absolute rotation values can serve as key criteria for identifying potential shear band locations.

(3) A shear band localization method is developed based on PDL-GAN data augmentation and PCA + Gaussian regional rotational angle prediction. The PDL-GAN model generates augmented samples consistent with the statistical characteristics of real MEMS rotational responses based on non-parametric probability density estimation and further improves data distribution consistency by removing anomalous samples that deviate from the real distribution using the Bray–Curtis distance. Comparative results show that the PCA + Gaussian model achieves superior overall performance in prediction accuracy, probabilistic forecasting, and physical interpretability, with MAE, RMSE, CRPS, Coverage95, and Interval Width values of 0.0803, 0.1024, 0.0801, 0.9773, and 3.7883, respectively.

Under different loading stages, the predicted regional rotational fields produced by the PCA + Gaussian model effectively capture the high-rotation concentration zone beneath the slope crest and the evolution trend of shear band propagation into the slope interior, with the predicted shear band locations generally consistent with the experimental observations in terms of the overall trajectory. Compared with DEM simulations, the PCA + Gaussian model does not require repeated and computationally expensive particle contact calculations after training and can rapidly generate regional rotational fields and shear band locations. This demonstrates that the proposed method provides an efficient data-driven approach for the rapid identification and early warning of progressive failure in gravel–soil slopes.

As a preliminary investigation, this study verifies the feasibility of integrating MEMS rotational responses with machine learning methods based on laboratory-scale model tests and DEM simulation data. However, the transition from laboratory-scale validation to field-scale engineering applications still involves several challenges. The influence of sensor deployment density, optimal data acquisition frequency, and geological variability on rotational response characteristics and shear band identification accuracy requires further investigation. Future work should expand the experimental conditions by considering different slope angles, water contents, loading schemes, and gradation characteristics, and incorporate field monitoring data for model calibration and validation. Moreover, adaptive sensor layout strategies and robust prediction frameworks under complex geological environments should be developed to enhance the applicability of the proposed method for shear band identification and instability early warning in real engineering slopes. Furthermore, the proposed MEMS-based rotational response analysis framework may be integrated with failure-time prediction approaches, such as the Fukuzono inverse velocity method, in future studies to provide a more comprehensive early-warning framework combining the spatial localization of internal shear bands with the temporal prediction of slope instability.

## Figures and Tables

**Table 1:** Test Parameters of T1–T3 Test Groups.

Groups	Red Clay: Gravel (%)	Density (g/cm^3^)	Moisture Content (%)
T1	100: 0	1.71	19.1
T2	80: 20	1.91	19.1
T3	50: 50	2.11	19.1

**Table 2:** Macroscopic Mechanical Parameters of Gravelly Soil.

Groups	*φ* (°)	$c_{app}$ (kPa)	*μ*
T1	6.3	30.1	0.11
T2	11.3	31.1	0.20
T3	11.9	40.0	0.21

**Table 3:** Microscopic Contact Parameters for DEM Simulations.

Groups	Contact Modulus E (MPa)	Particle Density ρ (g/cm^3^)	Particle Friction Coefficient $\mu_{P}$	Normal-to-Shear Stiffness Ratio *k_n_*/*k_s_*	Damping Coefficient D
T1	100	1.71	0.11	1	0.4
T2	200	1.91	0.20	1	0.4
T3	200	2.11	0.21	1	0.4

**Table 4:** Main Hyperparameter Settings of Each Model.

Models	Key Parameters	Value of the Parameter
Baseline	Coefficient of regularization *α*	0.1
Baseline	Form of loss	Robust regression loss
PCA + Gaussian	Number of principal components *K*	5
PCA + Gaussian	Selection principle of principal components	The cumulative variance contribution rate reached 95%
PCA + Gaussian	Form of probability output	Predicted mean and 95% confidence interval
XGBoost	Maximum tree depth	3
XGBoost	Number of trees	100
XGBoost	Learning rate	0.05
XGBoost	Methods of training and control	Early stop strategy based on validation set error
VAE	Hidden layer dimension	128
VAE	Latent variable dimension	16
VAE	Learning rate	1 × 10^−3^
Transformer	Number of encoder layers	2
Transformer	Attention head count	4
Transformer	Hidden Dimensions	128
Transformer	Dropout	0.1

**Table 5:** Comparison of Regional Rotation-Angle Prediction Performance of Different Machine Learning Models.

Models	MAE	RMSE	CRPS	Coverage95	Interval Width
Transformer	0.1877	0.2566	0.1877	0.9966	3.6253
VAR	0.1453	0.2004	0.1453	0.9874	4.1999
XGBoost	0.0926	0.1302	0.0926	0.9769	3.7539
PCA + Gaussian	0.0803	0.1024	0.0801	0.9773	3.7883
Baseline	0.0801	0.1004	0.0801	0.9773	3.7885

## Data Availability

The data presented in this study are available from the corresponding author upon reasonable request. The data are not publicly available because they are part of an ongoing research project.

## Abbreviations

The following abbreviations are used in this manuscript:

MEMS	Micro-Electro-Mechanical Systems
IMU	Inertial Measurement Unit
MEMS-IMU	Micro-Electro-Mechanical Systems Inertial Measurement Unit
DEM	Discrete Element Method
PDL-GAN	Probability Density Layer–Generative Adversarial Network
PCA	Principal Component Analysis
AHRS	Attitude and Heading Reference System
TDR	Time-Domain Reflectometry
GNSS	Global Navigation Satellite System
InSAR	Interferometric Synthetic Aperture Radar
CAD	Computer-Aided Design
FIJI	Fiji Is Just ImageJ
TIFF	Tagged Image File Format
PDL	Probability Density Layer
GAN	Generative Adversarial Network
XGBoost	Extreme Gradient Boosting
VAR	Vector Autoregression
VAE	Variational Autoencoder
MAPE	Mean Absolute Percentage Error
MAE	Mean Absolute Error
RMSE	Root Mean Square Error
CRPS	Continuous Ranked Probability Score
CDF	Cumulative Distribution Function
B–C	Bray–Curtis Distance
RMS	Root Mean Square
TTL	Transistor–Transistor Logic
RAD	Radian
PCA + Gaussian	Principal Component Analysis + Gaussian Model
